# Physiological Adjustments and Circulating MicroRNA Reprogramming Are Involved in Early Acclimatization to High Altitude in Chinese Han Males

**DOI:** 10.3389/fphys.2016.00601

**Published:** 2016-12-02

**Authors:** Bao Liu, He Huang, Shou-Xian Wang, Gang Wu, Gang Xu, Bing-Da Sun, Er-Long Zhang, Yu-Qi Gao

**Affiliations:** ^1^Key Laboratory of High Altitude Environmental Medicine, Third Military Medical University, Ministry of EducationChongqing, China; ^2^Key Laboratory of High Altitude Medicine, PLAChongqing, China; ^3^Key Laboratory of High Altitude Medicine, Institute of Medicine and Hygienic Equipment for High Altitude Region, College of High Altitude Military Medicine, Third Military Medical University, Ministry of Education of ChinaChongqing, China

**Keywords:** acclimatization, high altitude, hypoxia, circulating microRNAs, Chinese Han males

## Abstract

**Background:** Altitude acclimatization is a physiological process that restores oxygen delivery to the tissues and promotes oxygen use under high altitude hypoxia. High altitude sickness occurs in individuals without acclimatization. Unraveling the molecular underpinnings of altitude acclimatization could help understand the beneficial body responses to high altitude hypoxia as well as the altered biological events in un-acclimatized individuals. This study assessed physiological adjustments and circulating microRNA (cmiRNA) profiles in individuals exposed to high altitude, aiming to explore altitude acclimatization in humans.

**Methods:** Ninety volunteers were enrolled in this study. Among them, 22 individuals provided samples for microRNA arrays; 68 additional individuals constituted the validation set. Un-acclimatized individuals were identified by the Lake Louise Scoring System. Thirty-three phenotypes were recorded pre- and post-exposure to high altitude, including stress hormones, lipid profiles, hematological indices, myocardial enzyme spectrum, and liver and kidney function related enzymes. CmiRNA expression profiles were assessed using miRCURYTM LNA Array (v.18.0) screening, with data validated by quantitative reverse-transcription polymerase chain reaction (qRT-PCR). Then, associations of plasma microRNA expression with physiological adjustments were evaluated. The biological relevance of the main differentially expressed cmiRNAs was explored by bioinformatics prediction.

**Results:** Nineteen of the 33 phenotypes were significantly altered during early altitude acclimatization, including hematological indices, lipid profiles, and stress hormones; meanwhile, 86 cmiRNAs (79 up-regulated and 7 down-regulated) showed differential expression with statistical significance. Among them, 32 and 25 microRNAs were strongly correlated with low-density lipoprotein-cholesterol and total cholesterol elevations, respectively. In addition, 22 microRNAs were closely correlated with cortisol increase. In un-acclimatized individuals, 55 cmiRNAs were up-regulated and 36 down-regulated, compared with acclimatized individuals. The HIF signaling pathway was suppressed in un-acclimatized individuals.

**Conclusion:** Physiological adjustments, including the hematological system, stress hormones, and lipid molecules contributed to early altitude acclimatization, and showed strong correlations with cmiRNA reprogramming. Moreover, acclimatized and un-acclimatized individuals showed different cmiRNA profile. Suppression of the HIF-1 signaling pathway by microRNA regulation may play a key role in the pathogenesis of un-acclimatization with high altitude hypoxia.

## Introduction

High altitudes support considerable populations worldwide, including those living in highland areas and individuals who flock to plateaus for outdoor activities. During exposure to high altitude, certain physiological, and biochemical changes occur for high altitude adaptation. However, acute exposure to high altitude hypoxia can cause a spectrum of disorders encompassing acute mountain sickness, high altitude pulmonary edema, and high altitude cerebral edema in un-acclimatized individuals (Basnyat and Murdoch, 2003; Bärtsch and Swenson, 2013). Altitude acclimatization is a physiological process taking place in the body upon exposure to hypoxia at high altitude. It comprises a number of responses by different body systems to restore oxygen delivery to tissues, promoting oxygen use despite a low partial pressure. The most important changes are increase in breathing (Robbins, 2007) and hemoglobin concentration (Bärtsch and Saltin, 2008). In addition, the hormonal and metabolic systems are influenced by high altitude hypoxia (Richalet et al., 2010; Hill et al., 2011). However, the mechanisms involved in altitude acclimatization have not been well explored at the molecular level.

MicroRNAs are small, noncoding RNAs that regulate gene expression post-transcriptionally, through RNA interference by targeting mRNAs at the 3′ or 5′ untranslated regions, or even the coding sequences (Lagos-Quintana et al., 2001; Bartel, 2004). Their regulatory roles have been demonstrated in various physiological and pathophysiological cellular processes such as proliferation (Lenkala et al., 2014), differentiation (Shivdasani, 2006), metabolism (Dumortier et al., 2013), and apoptosis (Su et al., 2015). Therefore, circulating microRNA (cmiRNA) expression profiles are being intensively assessed for their functions in various pathogenic processes (Mitchell et al., 2008; Creemers et al., 2012; Wang et al., 2015). These extracellular microRNAs are considered a novel group of signaling molecules, and act as tools in cell-to-cell communication that is necessary for cells to coordinate and execute biological functions, and perform specialized tasks (Mittelbrunn et al., 2011; Kosaka et al., 2012). Moreover, a few reports have shown that hypoxia is an important proximal regulator of microRNA biogenesis and function (Nallamshetty et al., 2013; Rupaimoole et al., 2014). Recently, a study assessing Tibetans and migrant Han Chinese found that these two populations present different circulating microRNA profiles compared with lowlanders, and suggested that cmiRNAs play key roles in high altitude adaptation (Yan et al., 2015). Therefore, we hypothesized that cmiRNA profiles of individuals exposed to high altitude acutely would be altered by high altitude hypoxia, with cmiRNA reprogramming exerting a pivotal role in early altitude acclimatization and participating in the pathogenesis of un-acclimatization.

Therefore, in the present study, we performed miRCURYTM LNA Array (v.18.0) (Exiqon) screening, followed by data validation with quantitative reverse-transcription polymerase chain reaction (qRT-PCR) to evaluate plasma microRNA profiles of individuals before and after exposure to high altitude. In addition, we analyzed the associations of altered plasma microRNAs with physiological and biochemical characteristics. Moreover, we explored the potential role of cmiRNAs in un-acclimatization at high altitude. Interestingly, our data indicated that CmiRNA reprogramming regulates early altitude acclimatization and participates in the pathogenesis of un-acclimatization.

## Methods

### Participants and plasma sample processing

Excluding individuals with a history of smoking, 90 young male volunteers (age, 23 ± 3.7 years) acutely ascended to Lhasa (3648 m), China, by train within 48 h, were included in this study. All volunteers had the same diet during the investigation. Among them, 22 individuals were randomly selected for microRNA array screening, while the remaining 68 constituted the validation set (Table 1). All blood samples were collected in EDTA tubes using standard operating procedures. The study protocol was approved by the ethics committees of third Military Medical University, China, in accordance with the Declaration of Helsinki; all participants provided written informed consent.

**Table 1:** **Characteristics of subjects**.

**Characteristics**	**Acclimatization**	**Un-acclimatization**	***P*-value**
**MICRORNAS ARRAY SCREENING SET (*n* = 22)**			
**Age (year)**			
Mean	28.1 ± 3.8	25.7 ± 2.1	
Range	24–35	24–32	
**Sex**			
Male	9	13	
Female	0	0	
BMI(kg/m^2^)	22.6 ± 2.2	21.9 ± 1.7	0.475
LLS	2.8 ± 1.2	6.0 ± 1.2	<0.001
Headache severity	1.0 ± 0.0	1.5 ± 0.7	0.027
**VALIDATION SET (*n* = 68)**			
**Age (year)**			
Mean	20.1 ± 3.0	23.1 ± 3.1	
Range	17–27	18–28	
**Sex**			
Male	31	37	
Female	0	0	
BMI(kg/m^2^)	20.9 ± 2.3	21.9 ± 1.7	0.058
LLS	0.9 ± 1.2	6.1 ± 1.6	<0.001
Headache severity	0.1 ± 0.3	1.8 ± 0.9	<0.001

### Data collection

Blood samples from volunteers were collected in the morning at sea level, and 2 days after exposure to high altitude. Self-assessment questionnaires of the Lake Louise Scoring System and main parameters, including blood pressure, heart rate, and blood oxygen saturation, were recorded prior to departure, and at each day during high altitude exposure (7 days).

Thirty-three phenotypes were recorded (Supplementary Table 1), including stress hormones, lipid profiles, hematological indices, myocardial enzyme spectrum, and liver and kidney function related enzymes. High performance liquid chromatography was carried out to detect blood catecholamine amounts, and chemiluminescent immunometeic assay was used to measure adrenocorticotropic hormone and cortisol levels. Serum concentrations of C-reactive protein and biochemical indexes were assessed by immunonephelometry and on a Roche Chemistry Analyzer, respectively. Hematological indices, including red blood cell, hematocrit, hemoglobin, white blood cell and platelet levels, were evaluated on an XE-2100 analyzer (Sysmex, Kobe, Japan) with commercial reagents, immediately after blood collection.

### Evaluation of acclimatization at high altitude

Acclimatization of volunteers at high altitude was evaluated by using the Lake Louise Scoring System (LLS), which comprises a questionnaire and a scorecard that determine severity. During the 7 day study period, individuals with LLS ≥ 3 (including a headache score ≥ 1), at least once, were considered to be un-acclimatized, while those without headache or showing LLS <3 were considered to have a better acclimatization at high altitude.

### RNA isolation and microRNA array analysis

Total RNA was isolated using TRIzol (Invitrogen) and purified with RNeasy mini kit (QIAGEN) according to manufacturers' instructions. RNA quality and quantity were measured on a NanoDrop spectrophotometer (ND-1000, Nanodrop Technologies). Detailed RNA quality and quantity data are listed in Supplementary Table 2. After quality control, the miRCURY™ Hy3™/Hy5™ Power labeling kit (Exiqon, Vedbaek, Denmark) was used according to the manufacturer's guideline for microRNA labeling. Then, Hy3™-labeled samples were hybridized on a miRCURYTM LNA Array (v.18.0) (Exiqon), according to the manufacturer's protocols. Following hybridization, the slides were washed several times with a Wash buffer kit (Exiqon), and scanned on an Axon GenePix 4000B microarray scanner (Axon Instruments, Foster City, CA). Scanned images were then imported into the GenePix Pro 6.0 software (Axon) for grid alignment and data extraction. Replicated microRNAs were averaged, and probes with intensities ≥30 in all samples were selected for normalization. After normalization, significantly differentially expressed microRNAs between the two groups were identified using Fold change and *P*-value cutoffs of 2 and 0.05, respectively.

### Quantitative reverse-transcription polymerase chain reaction (qRT-PCR)

For qRT-PCR, a synthetic *Caenorhabditis elegans* microRNA, cel-miR-39 (Qiagen, Valencia, CA, USA), was added to plasma samples as a control prior to RNA extraction. Total RNA was extracted from 200 μL individual plasma samples with a miRNeasy extraction kit (Qiagen, Valencia, CA, USA) according to the manufacturer's instructions. RNA (6 μl) was reverse transcribed into cDNA (in a final volume of 10 μl) using a reverse transcription kit (GenePharma, Shanghai, China). Subsequently, quantitative real-time PCR was performed on an iQ™5 Real-Time PCR Detection System (Bio-Rid, USA) using SYBR Green. The primers used for qRT-PCR are listed in Table 2. Relative microRNA levels were determined by the ΔΔCT method.

**Table 2:** **Primers used for qRT-PCR verification of differently expressed circulating microRNAs**.

**microRNA name**	**Forward primer**	**Reverse primer**
miR-181b-5p	CACGACACCAACATTCATTGC	TATGGTTGTTCTCGTCTCCTTCTC
miR-676-3p	ACGCCGTCCTGAGGTTGT	TATGGTTTTGACGACTGTGTGAT
miR-193b-5p	AGGCCGGGGTTTTGAGG	TATGGTTGTTCACGACTCCTTCAC
miR-3591-3p	GCCGCTTAAACACCATTGTC	TATGCTTGTTCTCGTCTCTGTGTC
cel-miR-39	ATATCATCTCACCGGGTGTAAATC	TATGGTTTTGACGACTGTGTGAT

### Identifying potential functions of altered micrornas by bioinformatics

After microRNA profiling, we searched three well-known target prediction databases, including TargetScan (http://www.targetscan.org) (Lewis et al., 2003), miRanda (http://www.microrna.org) (Enright et al., 2003) and miRBase (http://www.mirbase.org/) (Griffiths-Jones, 2004), to identify mRNA targets of the altered microRNAs. To reduce the chance of false positives, only the targets obtained in all three programs were considered putative candidates for Gene Ontology (GO) (Ashburner et al., 2000) and KEGG pathway analysis (Kanehisa et al., 2008).

### Statistical analysis

Normality was assessed for all datasets by the Shapiro-Wilk's test. Then, statistically significant differences in phenotypes were identified by paired-samples *t*-test or Wilcoxon signed-rank test. The Pearson coefficient correlation method was used for correlation analyses between phenotypes and microRNA expression. Independent *t*-test or Mann-Whitney U test was carried out to assess phenotypic differences between acclimatized and un-acclimatized individuals. Areas under the receiver-operating characteristic curves (AUCs) were evaluated using Swets classification (Gasparini et al., 2015): AUC = 0.5, no diagnostic value; 0.5 < AUC <0.7, accuracy to only a small degree; 0.7 < AUC <0.9, fair accuracy; 0.9 < AUC ≤ 1, high accuracy. *P* < 0.05 was considered statistically significant. All statistical analyses were performed with the R software (version 3.2.3, R Foundation for Statistical Computing, Vienna, Austria) using the “gmodels” package for principle component analysis (PCA). The suitability of PCA was assessed prior to analysis using the Kaiser-Meyer-Olkin and Bartlett's test.

## Results

### Phenotypes with statistically significant differences post- and pre-exposure to high altitude

**Figure 1:**
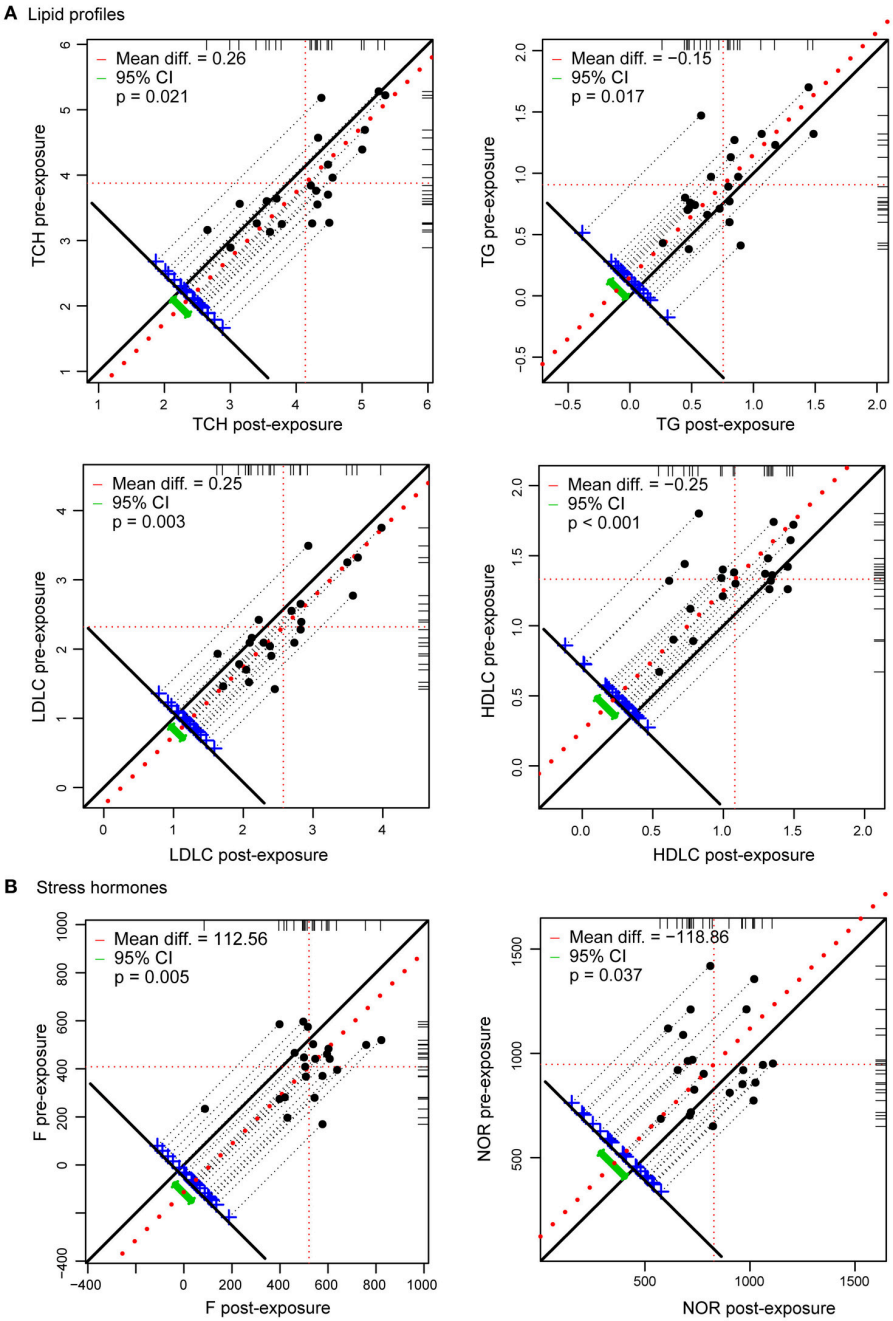
**Ascent to high altitude is associated with adjustments of lipid profiles and stress hormones (***n*** = 22). (A)** Lipid profiles were changed during the exposure to high altitude. TC and LDLC were increased, while TG and HDLC were decreased. **(B)** Effects of hypoxia on stress hormones are expressed as differences in plasma concentration of F and NOR at the indicated altitude from normoxic concentrations at sea level.

Exposure of healthy lowlanders to high altitude was accompanied by progressively increased heart rate and blood pressure during the first week at high altitude (Supplementary Figure 1), which are expected cardiovascular adjustments to the unavoidable reduction in oxygen availability. Oxygen saturation of arterial hemoglobin decreased with the ascent due to a reduction in inspired oxygen partial pressure (Supplementary Figure 1). To identify the most dramatic phenotypic changes at high altitude, 33 parameters were examined, and 19 of them were significantly altered at high altitude compared with pre-exposure values (Figure 1; Supplementary Figure 2; Supplementary Table 3). Among these, hematological indices, including red blood cell, hematocrit and hemoglobin levels, were significantly increased after exposure to high altitude as expected. In addition, cortisol (relevant to stress) and triglyceride, total cholesterol, lactate dehydrogenase, high-density lipoprotein-cholesterol and low-density lipoprotein-cholesterol (involved in metabolism significantly) levels were significantly altered by high altitude hypoxia.

### Comparison of cmiRNA profile before and after exposure to high altitude

**Figure 2:**
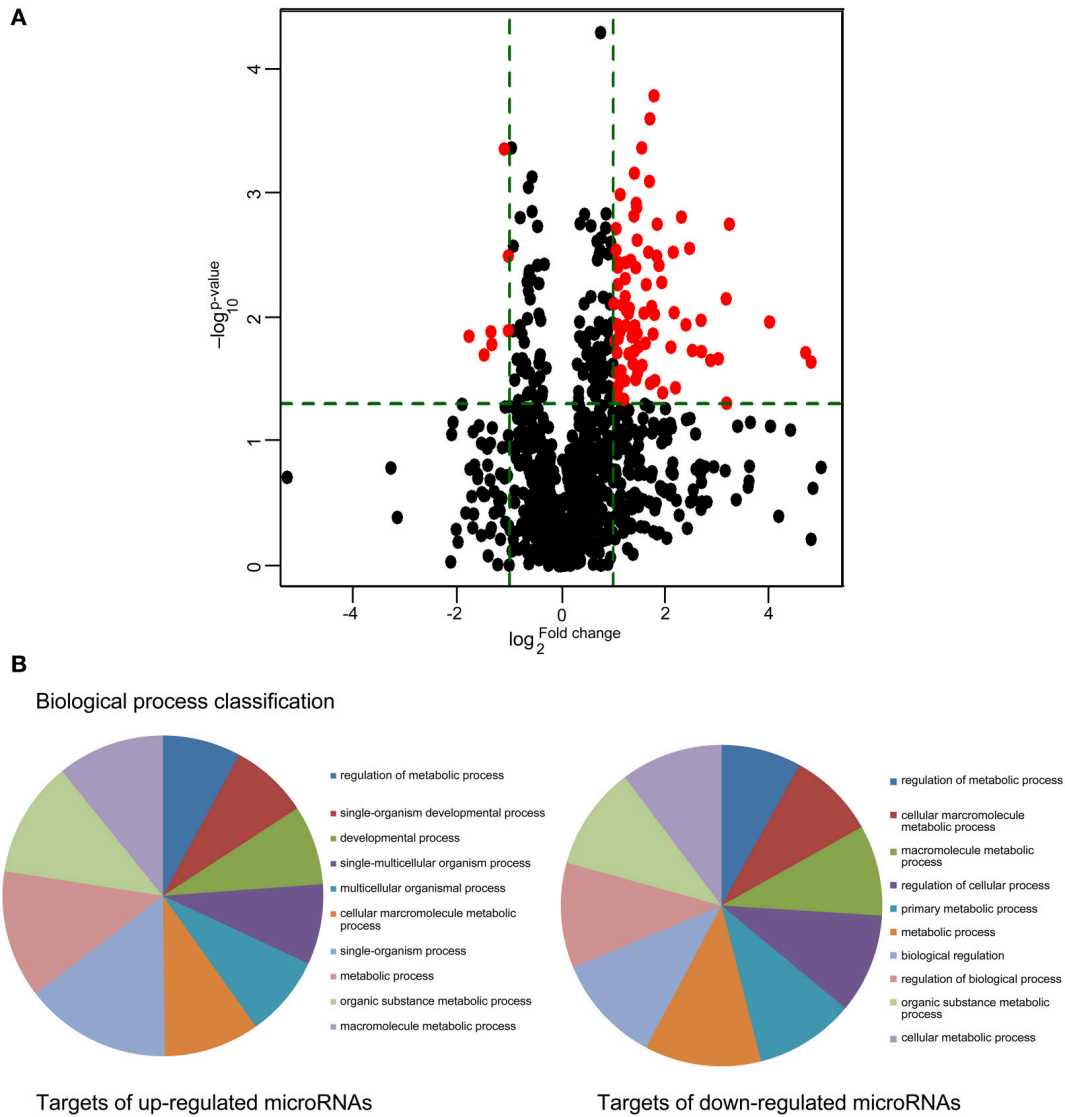
**Metabolism process was mainly regulated by differentially expressed cmiRNAs derived by high altitude hypoxia. (A)** Comparisons of all microRNAs in microarray analysis of RNA isolated from plasma of individuals before and after exposure to high altitude. The volcano plot displays the relationship between fold-change and significance using a scatter plot view. The red points in the plot represent the differentially expressed microRNAs with statistical significance. **(B)** PANTHER biological process classification. Pie chart of Gene Ontology distribution terms associated to genes regulated by differentially expressed cmiRNAs.

A microRNA expression array was utilized to examine circulating microRNA expression changes at high altitude. Of the 2383 probes assessed, 86 microRNAs (79 up-regulated and 7 down-regulated) showed differential expression with statistical significance, and were selected for subsequent analysis (Figure 2A).

Meanwhile, computational predictions of the target genes were performed for the top 10 upregulated and all downregulated miRNAs, and relevant Gene Ontology categories and signaling pathways for all the target genes were identified. A large number of targets were significantly enriched in several key signaling pathways implicated in macromolecule metabolism (Figure 2B).

### Correlation analyses between the 19 altered phenotypes and 86 significantly altered microRNAs

**Figure 3:**
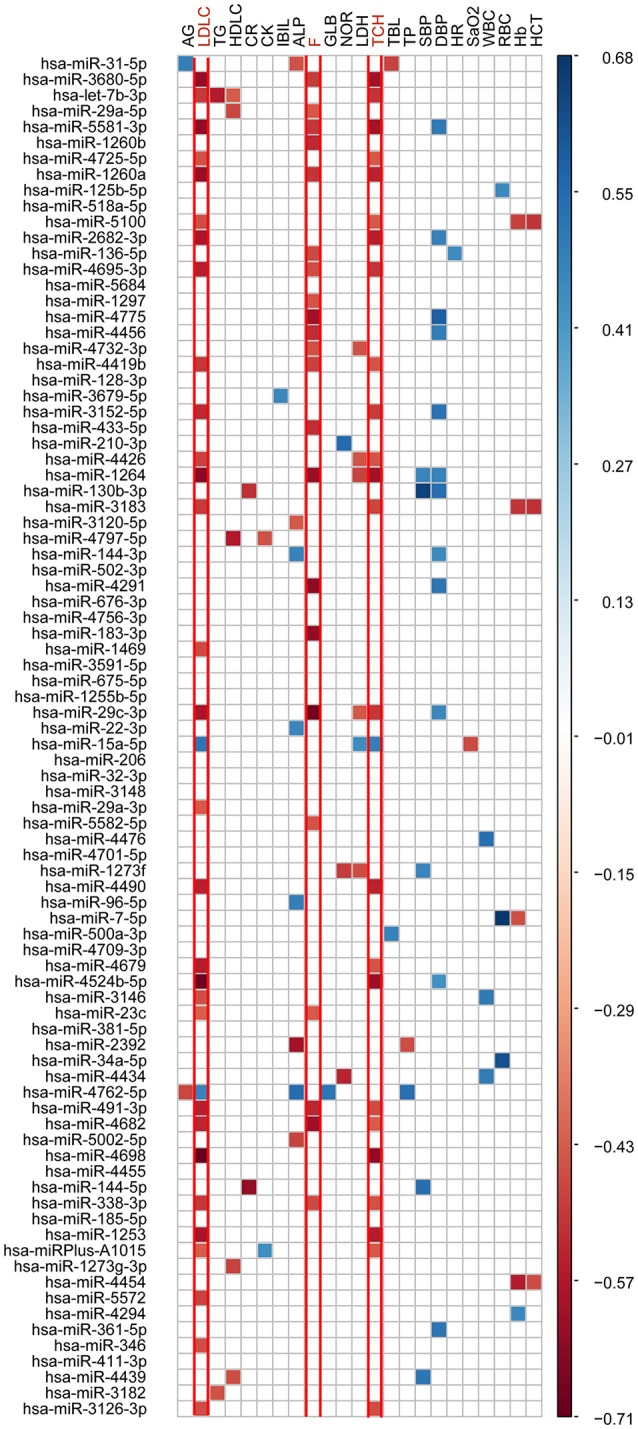
**Pearson correlation coefficient analyses of 19 significantly changed phenotypes and main signs with 86 significantly differentially expressed cmiRNAs**. The warm colors represent significant negative correlations, and the cold colors represent significant positive correlations. White indicates no significant association.

To further identify the cmiRNAs potentially responsible for the observed phenotypic changes, the associations of 19 significantly altered phenotypes with 86 robustly differentially expressed cmiRNAs were assessed. Of the 86 differentially expressed microRNAs, 67 were associated with the 19 significantly changed phenotypes at high altitude (Figure 3; Supplementary Table 4). Interestingly, the vast majority of microRNAs were substantially related to metabolic parameters and stress hormones. Importantly, 32 and 25 microRNAs were strongly associated with total cholesterol (TCH) and low-density lipoprotein-cholesterol (LDLC) elevations, respectively; meanwhile, 22 microRNAs were strongly correlated with increased cortisol (F).

### Phenotypes and cmiRNAs between acclimatized and un-acclimatized individuals

**Figure 4:**
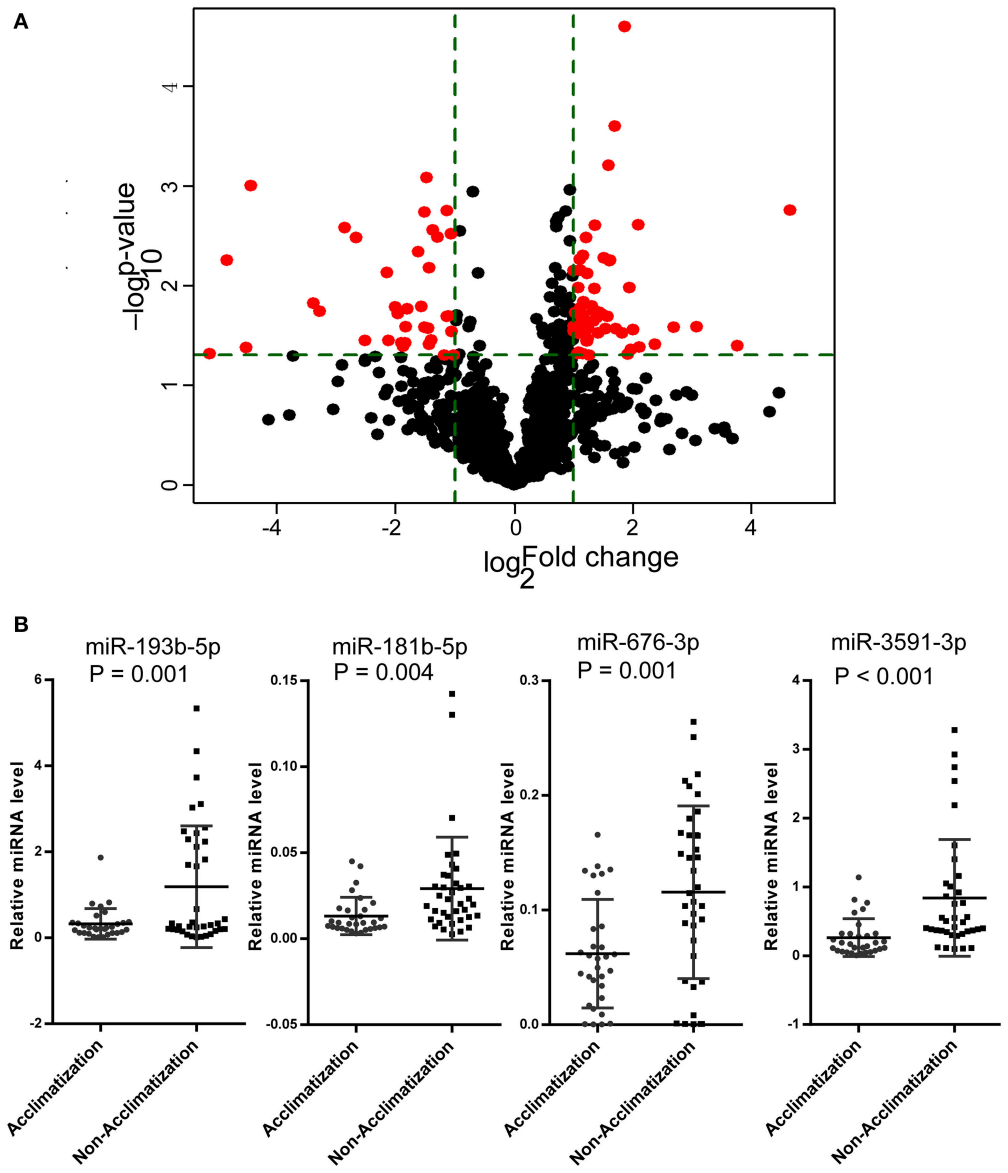
**CmiRNAs expression profile was different between acclimatized and un-acclimatized individuals. (A)** Comparisons of all microRNAs in microarray analysis of RNA isolated from plasma of acclimatized and un-acclimatized individuals. The volcano plot displays the relationship between fold-change and significance using a scatter plot view. The red points in the plot represent the differentially expressed microRNAs with statistical significance. **(B)** The relative concentrations of miR-181b-5p, miR-676-3p, miR-193b-5p, and miR-3591-3p in the plasma samples from the acclimatization (*n* = 31) and un-acclimatization (*n* = 37) groups in validation set.

**Figure 5:**
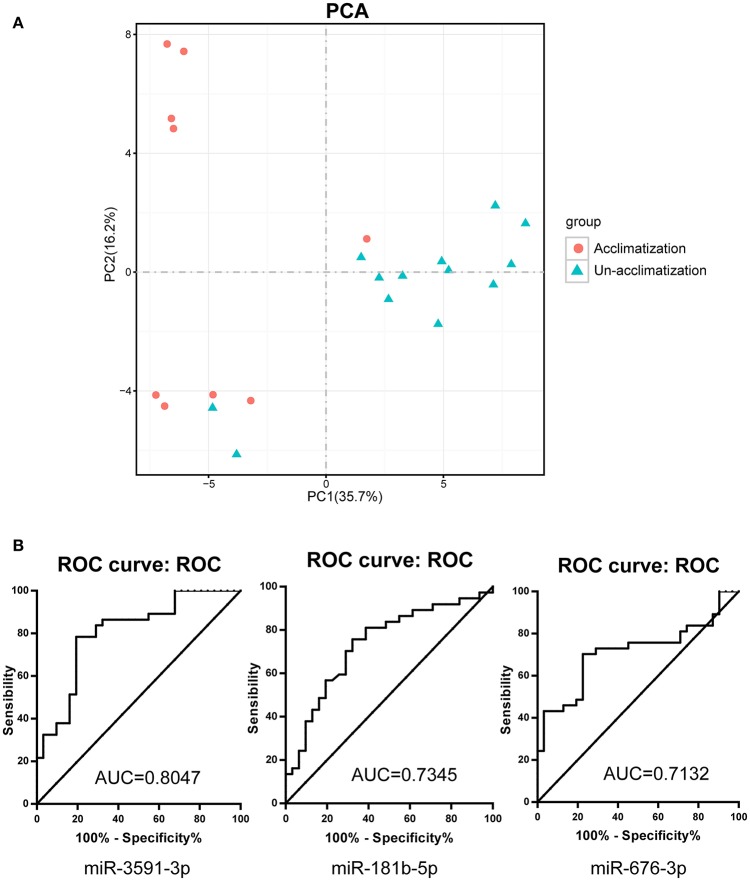
**Acclimatized and un-acclimatized individuals present different response to high altitude hypoxia. (A)** Principle component analysis (PCA) results revealing the impact of high altitude hypoxia on cmiRNAs expression between acclimatized and un-acclimatized individuals. **(B)** Receiver-operator characteristic (ROC) curve analyses for acclimatized and un-acclimatized individuals in validation set. AUC, area under receiver-operator characteristic curve.

Based on the LLS of all individuals in the microRNAs array screening set, 13 participants were considered un-acclimatized individuals with acute mountain sickness, whilst the remaining 9 had better acclimatization at high altitude. The phenotypic changes in acclimatization individuals presented no significant differences compared with un-acclimatized subjects (Supplementary Table 5). To ensure whether there are differences in circulating microRNA expression profiles between acclimatized and un-acclimatized individuals, we analyzed circulating microRNA expression profiles in both groups. Interestingly, 91 (55 up-regulated and 36 down-regulated) cmiRNAs were differentially expressed under the set criteria (*p* < 0.05 and fold change ≥2) (Figure 4A). Among the 91 differentially expressed cmiRNAs, there was an overlap of 11 cmiRNAs with the 86 cmiRNAs differentially expressed after exposure to high altitude. The suitability of PCA was assessed prior to analysis. Inspection of the correlation matrix showed that all variables had at least one correlation coefficient greater than 0.3. The overall Kaiser-Meyer-Olkin (KMO) score was 0.78, with individual KMO measures all above 0.7, classified as “middling” to “meritorious” according to Kaiser (1974). The Bartlett's test of sphericity was statistically significant (*p* < 0.0001), indicating that the data are likely factorizable. PCA further confirmed these results, showing that un-acclimatized individuals form a separate group from acclimatized individuals (Figure 5A).

To further explore the sensitivity and specificity of cmiRNAs in discriminating between acclimatized and un-acclimatized individuals, we measured the expression levels of has-miR-676-3p (MIMAT0018204), has-miR-3591-3p (MIMAT0019877), has-miR-181b-5p (MIMAT0000257) and has-miR-193b-5p (MIMAT0004767), the most differentially up-regulated cmiRNAs, by qRT-PCR. As shown in Figure 4B, qRT-PCR data were consistent with microarray findings. Indeed, has-miR-676-3p, has-miR-3591-3p, has-miR-181b-5p, and has-miR-193b-5p were differentially expressed between acclimatized and un-acclimatized individuals. Moreover, among the four cmiRNAs, has-miR-3591-3p, has-miR-676-3p, and has-miR-181b-5p yielded ROC AUCs of 0.805 (*P* < 0.0001), 0.713 (*P* < 0.01), and 0.734 (*P* < 0.01), respectively, indicating that these cmiRNAs were fairly accurate in determining un-acclimatization diagnosis (Figure 5B).

### Biological relevance of differentially expressed microRNAs between acclimatized and un-acclimatized individuals

To further assess the pathogenesis of un-acclimatization at high altitude hypoxia, GO and KEGG pathway analyses were performed to disclose functional enrichment of genes predicted to be regulated by the top 10 differentially up- and down-regulated cmiRNAs between acclimatized and un-acclimatized individuals. Interestingly, several signaling pathways were regulated by these cmiRNAs. Specifically, the HIF-1 signaling pathway was suppressed by cmiRNAs up-regulated in un-acclimatized individuals (Table 3).

**Table 3:** **KEGG pathway analysis results of the target genes of top 10 up-regulated circulating microRNAs in un-acclimatized individuals compared with acclimatization individuals (***P*** < 0.01)**.

**Pathway ID**	**Pathway name**	***P*-value**
hsa04066	HIF-1 signaling pathway	1.53E-03
hsa04024	cAMP signaling pathway	2.89E-03
hsa04010	MAPK signaling pathway	3.88E-03
hsa04064	NF-kappa B signaling pathway	4.89E-03

## Discussion

CmiRNAs are so remarkably stable that they can be specific to certain physiological and pathological conditions (Mitchell et al., 2008; Mi et al., 2013). The biological relevance of cmiRNAs is regarded as a global, hormone-like functional mechanism that might allow regulation of gene expression across tissues at a distance (Turchinovich et al., 2013). This study demonstrated that short-time high altitude hypoxia could elicit a significant change in cmiRNAs expression profiles. The expression levels of cmiRNAs showed strong linear correlations with plasma hormones and lipid profiles during human acclimatization to high altitude. Furthermore, un-acclimated individuals presented different cmiRNA profile compared with acclimated ones.

It has been previously reported that lipid metabolism (Young et al., 1989), stress hormones (Richalet, 2014), and the hematological system (Frisancho, 1975) are altered by high altitude exposure, although the molecular underpinnings of such changes remain unclear. This study revealed that cmiRNA expression showed strong correlations with such changes. CmiRNAs are extracellular microRNAs with excellent stability (Mitchell et al., 2008); they are small, protein noncoding RNAs that regulate gene expression post-transcriptionally, through RNA interference by targeting mRNAs at the 3′ or 5′ untranslated regions, or even the coding sequence (Huntzinger and Izaurralde, 2011). Multiple microRNAs are involved in lipid metabolism and stress response (Leung and Sharp, 2010; Fernández-Hernando et al., 2011). In this study, the expression of many circulating microRNAs showed significant correlations with the stress hormone F and lipid metabolism related products such as LDLC and TCH. The target genes of the top 10 upregulated and all downregulated microRNAs were involved in metabolism. F is a steroid hormone associated with gluconeogenesis, immune suppression, and fat and carbohydrate metabolism (Elenkov, 2004; Christiansen et al., 2007). LDLC and TCH as lipids, are risk factors for ischemic heart disease and stroke, which have a pathophysiology process of hypoxia (Anand, 2003; Writing Group et al., 2016). Metabolic adjustment is a key event in early altitude acclimatization. Moreover, it is intriguing that differentially expressed microRNAs between pre- and pro-exposure to high altitude overlapped with those found in ischemic stroke patients (Li et al., 2015). This indicates that hypoxia sensitive cmiRNAs are prevalent in hypoxia related diseases.

High altitude hypoxia could lead to complex clinical syndrome in individuals with poor acclimatization (Hackett and Roach, 2001). It is important to accurately evaluate acclimatization while engaging people in ascents to higher elevations or descent to plains, in order to decrease the morbidity of severe high altitude illness. In the past, the discrimination between acclimatized and un-acclimatized individuals was mainly based on clinical questionnaires (Bärtsch and Swenson, 2013). CmiRNAs are considered a novel group of signaling molecules, and have been applied successfully in the diagnosis of complex diseases (Mi et al., 2013; Pirola et al., 2015). In the current study, cmiRNAs displayed higher sensitivities and specificities than disease phenotypes in distinguishing acclimatized from un-acclimatized individuals. Indeed, single circulating microRNAs could discriminate un-acclimatized individuals from people exposed to high altitude with a fair accuracy. Several signaling pathways were regulated by the differentially expressed cmiRNAs. Among these, the HIF-1 signaling pathway promotes human adaptation to high altitude through the transcriptional regulation of its target genes, to induce a systemic increase of erythropoietin and regulate the metabolic process (Bigham and Lee, 2014). The cmiRNAs suppressing HIF-1 pathway genes were up-regulated in un-acclimatized individuals, which may prevent them from restoring hemostasis at high altitude. So, interfering the expression of these cmiRNAs may represent a great avenue to help people acclimatize to high altitude.

In this study, the sample size was relatively small because of high experimental costs. All volunteers were young men. There is a need for testing more individuals, also including females, other races and ages, whose cmiRNA profile were not assessed, to confirm our findings.

In conclusion, circulating microRNA expression profiles of individual exposed to high altitude acutely were assessed. Interestingly, cmiRNAs showed strong correlations with physiological adjustments, especially stress hormones and lipids. The genes predicted to be regulated by the differentially expressed microRNAs were enriched in metabolism. Moreover, cmiRNAs could be used for differentiating between acclimatized and un-acclimatized individuals, with higher sensitivity and specificity compared with phenotypes. The pathogenesis of un-acclimatization at high altitude hypoxia may be associated with inhibited HIF-1 signaling pathway through microRNA regulation.

## Availability of data and materials

All data have been submitted to GEO under the accession GSE89486.

## Author contributions

YG conceived and designed the study. HH and SW oversaw laboratory analyses and BL provided the overall supervision of the study. GW, EZ, and GX did the laboratory experiments or contributed the statistical analysis, or both GW and BS contributed to sample and physical data collections. BL drafted the report. All authors contributed to the interpretation of results, critical revision of the manuscript and approved the final manuscript. YG is the guarantor.

### Conflict of interest statement

The authors declare that the research was conducted in the absence of any commercial or financial relationships that could be construed as a potential conflict of interest.

## Abbreviations

cmiRNAs: circulating microRNAs
qRT-PCR: quantitative reverse-transcription polymerase chain reaction
LLS: Lake Louise Scoring System
GO: Gene Ontology
AUC: area under the receiver-operating characteristic curve
TCH: total cholesterol
LDLC: low-density lipoproteincholesterol
F: cortisol.

## References

[B1] Anand S. S. (2003). Quantifying effect of statins on low density lipoprotein cholesterol, ischaemic heart disease, and stroke: systematic review and meta-analysis. Law M. R., Wald, N. J., Rudnicka A. R. BMJ 326:1423. Vasc. Med. 8, 289–290. 10.1136/bmj.326.7404.1423 12829554 PMC162260

[B2] Ashburner M. Ball C. A. Blake J. A. Botstein D. Butler H. Cherry J. M.. (2000). Gene ontology: tool for the unification of biology. The gene ontology consortium. Nat. Genet. 25, 25–29. 10.1038/75556 10802651 PMC3037419

[B3] Bartel D. P. (2004). MicroRNAs: genomics, biogenesis, mechanism, and function. Cell 116, 281–297. 10.1016/S0092-8674(04)00045-5 14744438

[B4] Bärtsch P. Saltin B. (2008). General introduction to altitude adaptation and mountain sickness. Scand. J. Med. Sci. Sports 18(Suppl. 1), 1–10. 10.1111/j.1600-0838.2008.00827.x 18665947

[B5] Bärtsch P. Swenson E. R. (2013). Clinical practice: acute high-altitude illnesses. N. Engl. J. Med. 368, 2294–2302. 10.1056/NEJMcp1214870 23758234

[B6] Basnyat B. Murdoch D. R. (2003). High-altitude illness. Lancet 361, 1967–1974. 10.1016/S0140-6736(03)13591-X 12801752

[B7] Bigham A. W. Lee F. S. (2014). Human high-altitude adaptation: forward genetics meets the HIF pathway. Genes Dev. 28, 2189–2204. 10.1101/gad.250167.114 25319824 PMC4201282

[B8] Christiansen J. J. Djurhuus C. B. Gravholt C. H. Iversen P. Christiansen J. S. Schmitz O.. (2007). Effects of cortisol on carbohydrate, lipid, and protein metabolism: studies of acute cortisol withdrawal in adrenocortical failure. J. Clin. Endocrinol. Metab. 92, 3553–3559. 10.1210/jc.2007-0445 17609300

[B9] Creemers E. E. Tijsen A. J. Pinto Y. M. (2012). Circulating microRNAs: novel biomarkers and extracellular communicators in cardiovascular disease? Circ. Res. 110, 483–495. 10.1161/CIRCRESAHA.111.247452 22302755

[B10] Dumortier O. Hinault C. Van Obberghen E. (2013). MicroRNAs and metabolism crosstalk in energy homeostasis. Cell Metab. 18, 312–324. 10.1016/j.cmet.2013.06.004 23850315

[B11] Elenkov I. J. (2004). Glucocorticoids and the Th1/Th2 balance. Ann. N.Y. Acad. Sci. 1024, 138–146. 10.1196/annals.1321.010 15265778

[B12] Enright A. J. John B. Gaul U. Tuschl T. Sander C. Marks D. S. (2003). MicroRNA targets in Drosophila. Genome Biol. 5:R1. 10.1186/gb-2003-5-1-r1 14709173 PMC395733

[B13] Fernández-Hernando C. Suárez Y. Rayner K. J. Moore K. J. (2011). MicroRNAs in lipid metabolism. Curr. Opin. Lipidol. 22, 86–92. 10.1097/MOL.0b013e3283428d9d 21178770 PMC3096067

[B14] Frisancho A. R. (1975). Functional adaptation to high altitude hypoxia. Science 187, 313–319. 10.1126/science.1089311 1089311

[B15] Gasparini G. Vicini C. De Benedetto M. Salamanca F. Sorrenti G. Romandini M.. (2015). Diagnostic accuracy of obstructive airway adult test for diagnosis of obstructive sleep apnea. Biomed Res. Int. 2015:915185. 10.1155/2015/915185 26636102 PMC4618120

[B16] Griffiths-Jones S. (2004). The microRNA registry. Nucleic Acids Res. 32, D109–D111. 10.1093/nar/gkh023 14681370 PMC308757

[B17] Hackett P. H. Roach R. C. (2001). High-altitude illness. N. Engl. J. Med. 345, 107–114. 10.1056/NEJM200107123450206 11450659

[B18] Hill N. E. Stacey M. J. Woods D. R. (2011). Energy at high altitude. J. R. Army Med. Corps 157, 43–48. 10.1136/jramc-157-01-08 21465910

[B19] Huntzinger E. Izaurralde E. (2011). Gene silencing by microRNAs: contributions of translational repression and mRNA decay. Nat. Rev. Genet. 12, 99–110. 10.1038/nrg2936 21245828

[B20] Kaiser H. F. (1974). An index of factorial simplicity. Psychometrika 39, 32–36.

[B21] Kanehisa M. Araki M. Goto S. Hattori M. Hirakawa M. Itoh M.. (2008). KEGG for linking genomes to life and the environment. Nucleic Acids Res. 36, D480–D484. 10.1093/nar/gkm882 18077471 PMC2238879

[B22] Kosaka N. Iguchi H. Yoshioka Y. Hagiwara K. Takeshita F. Ochiya T. (2012). Competitive interactions of cancer cells and normal cells via secretory microRNAs. J. Biol. Chem. 287, 1397–1405. 10.1074/jbc.M111.288662 22123823 PMC3256909

[B23] Lagos-Quintana M. Rauhut R. Lendeckel W. Tuschl T. (2001). Identification of novel genes coding for small expressed RNAs. Science 294, 853–858. 10.1126/science.1064921 11679670

[B24] Lenkala D. LaCroix B. Gamazon E. R. Geeleher P. Im H. K. Huang R. S. (2014). The impact of microRNA expression on cellular proliferation. Hum. Genet. 133, 931–938. 10.1007/s00439-014-1434-4 24609542 PMC4677487

[B25] Leung A. K. Sharp P. A. (2010). MicroRNA functions in stress responses. Mol. Cell 40, 205–215. 10.1016/j.molcel.2010.09.027 20965416 PMC2996264

[B26] Lewis B. P. Shih I. H. Jones-Rhoades M. W. Bartel D. P. Burge C. B. (2003). Prediction of mammalian microRNA targets. Cell 115, 787–798. 10.1016/S0092-8674(03)01018-3 14697198

[B27] Li P. Teng F. Gao F. Zhang M. Wu J. Zhang C. (2015). Identification of circulating microRNAs as potential biomarkers for detecting acute ischemic stroke. Cell. Mol. Neurobiol. 35, 433–447. 10.1007/s10571-014-0139-5 25410304 PMC11486203

[B28] Mi S. Zhang J. Zhang W. Huang R. S. (2013). Circulating microRNAs as biomarkers for inflammatory diseases. MicroRNA 2, 63–71. 10.2174/2211536611302010007 25019052 PMC4092001

[B29] Mitchell P. S. Parkin R. K. Kroh E. M. Fritz B. R. Wyman S. K. Pogosova-Agadjanyan E. L.. (2008). Circulating microRNAs as stable blood-based markers for cancer detection. Proc. Natl. Acad. Sci. U.S.A. 105, 10513–10518. 10.1073/pnas.0804549105 18663219 PMC2492472

[B30] Mittelbrunn M. Gutiérrez-Vázquez C. Villarroya-Beltri C. González S. Sánchez-Cabo F. González M. A.. (2011). Unidirectional transfer of microRNA-loaded exosomes from T cells to antigen-presenting cells. Nat. Commun. 2:282. 10.1038/ncomms1285 21505438 PMC3104548

[B31] Nallamshetty S. Chan S. Y. Loscalzo J. (2013). Hypoxia: a master regulator of microRNA biogenesis and activity. Free Radic. Biol. Med. 64, 20–30. 10.1016/j.freeradbiomed.2013.05.022 23712003 PMC3762925

[B32] Pirola C. J. Fernández Gianotti T. Castaño G. O. Mallardi P. San Martino J. Mora Gonzalez Lopez Ledesma M.. (2015). Circulating microRNA signature in non-alcoholic fatty liver disease: from serum non-coding RNAs to liver histology and disease pathogenesis. Gut 64, 800–812. 10.1136/gutjnl-2014-306996 24973316 PMC4277726

[B33] Richalet J.-P. (2014). Endocrine function, in High Altitude, eds Swenson E. R. Bärtsch P. (New York, NY: Springer), 237–248.

[B34] Richalet J. P. Letournel M. Souberbielle J. C. (2010). Effects of high-altitude hypoxia on the hormonal response to hypothalamic factors. Am. J. Physiol. Regul. Integr. Comp. Physiol. 299, R1685–R1692. 10.1152/ajpregu.00484.2010 20926759

[B35] Robbins P. A. (2007). Role of the peripheral chemoreflex in the early stages of ventilatory acclimatization to altitude. Respir. Physiol. Neurobiol. 158, 237–242. 10.1016/j.resp.2007.03.008 17434348

[B36] Rupaimoole R. Wu S. Y. Pradeep S. Ivan C. Pecot C. V. Gharpure K. M.. (2014). Hypoxia-mediated downregulation of miRNA biogenesis promotes tumour progression. Nat. Commun. 5:5202. 10.1038/ncomms6202 25351346 PMC4215647

[B37] Shivdasani R. A. (2006). MicroRNAs: regulators of gene expression and cell differentiation. Blood 108, 3646–3653. 10.1182/blood-2006-01-030015 16882713 PMC1895474

[B38] Su Z. Yang Z. Xu Y. Chen Y. Yu Q. (2015). MicroRNAs in apoptosis, autophagy and necroptosis. Oncotarget 6, 8474–8490. 10.18632/oncotarget.3523 25893379 PMC4496162

[B39] Turchinovich A. Samatov T. R. Tonevitsky A. G. Burwinkel B. (2013). Circulating miRNAs: cell-cell communication function? Front. Genet. 4:119. 10.3389/fgene.2013.00119 23825476 PMC3695387

[B40] Wang J. Yu J. T. Tan L. Tian Y. Ma J. Tan C. C.. (2015). Genome-wide circulating microRNA expression profiling indicates biomarkers for epilepsy. Sci. Rep. 5:9522. 10.1038/srep09522 25825351 PMC4379481

[B41] Writing Group Members Mozaffarian D. Benjamin E. J. Go A. S. Arnett D. K. Blaha M. J.. (2016). Executive summary: heart disease and stroke statistics–2016 update: a report from the american heart association. Circulation 133, 447–454. 10.1161/CIR.0000000000000366 26811276

[B42] Yan Y. Shi Y. Wang C. Guo P. Wang J. Zhang C. Y.. (2015). Influence of a high-altitude hypoxic environment on human plasma microRNA profiles. Sci. Rep. 5:15156. 10.1038/srep15156 26468998 PMC4606833

[B43] Young P. M. Rose M. S. Sutton J. R. Green H. J. Cymerman A. Houston C. S. (1989). Operation Everest II: plasma lipid and hormonal responses during a simulated ascent of Mt. Everest. J. Appl. Physiol. 66, 1430–1435. 2651390 10.1152/jappl.1989.66.3.1430

