# Supplementary material for: Physiological Adjustments and Circulating MicroRNA Reprogramming Are Involved in Early Acclimatization to High Altitude in Chinese Han Males
Source: Front Physiol. 2016 Dec 2;7:601. doi: 10.3389/fphys.2016.00601 (PMC5133430; doi:10.3389/fphys.2016.00601)
Supplement: Supplementary file 1 [file Table1.DOCX]

Supplementary table 1 Abbreviation, Full Name and Unit of main Signs and 33 Phenotypes

| Abbreviation | Full Name | Unit |
| --- | --- | --- |
| SBP | systolic blood pressure | mmHg |
| DBP | diastolic blood pressure | mmHg |
| HR | heart rate | beat/min |
| SaO2 | blood oxygen saturation | % |
| NOR | norepinephrine | ng/L |
| ADR | adrenaline | ng/L |
| DOP | dopamine | ng/L |
| F | cortisol | nmol/L |
| ACTH | adrenocorticotropic hormone | ng/L |
| UA | uric acid | μmol/L |
| CR | creatinine | μmol/L |
| BUN | blood urea nitrogen | mmol/L |
| TP | total protein | g/L |
| ALB | albumin | g/L |
| GLB | globulin | g/L |
| A/G | albumin/globulin ratio | - |
| TBL | total bilirubin | μmol/L |
| DBL | direct bilirubin | μmol/L |
| IBIL | indirect bilirubin | μmol/L |
| AST | aspartate aminotransferase | U/L |
| ALT | alanine aminotransferase | U/L |
| R-GT | gamma-glutamyl transpeptidase | U/L |
| ALP | alkaline phosphatase | U/L |
| AST/ALT | aspartate aminotransferase/ alanine aminotransferase | - |
| CRP | C-reactive protein | mg/L |
| CK | creatine kinase | U/L |
| LDH | lactate dehydrogenase | U/L |
| CKMB | creatine kinase-MB | U/L |
| TCH | total cholesterol | mmol/L |
| TG | triglycerides | mmol/L |
| HDLC | high-density lipoprotein cholesterol | mmol/L |
| LDLC | low-density lipoprotein cholesterol | mmol/L |
| WBC | white blood cell | 10^9^/L |
| RBC | red blood cell | 10^12^/L |
| HG | hemoglobin | g/L |
| HCT | hematocrit | % |
| PLT | platelet | 10^9^/L |
