# Supplementary material for: Physiological Adjustments and Circulating MicroRNA Reprogramming Are Involved in Early Acclimatization to High Altitude in Chinese Han Males
Source: Front Physiol. 2016 Dec 2;7:601. doi: 10.3389/fphys.2016.00601 (PMC5133430; doi:10.3389/fphys.2016.00601)
Supplement: Supplementary file 2 [file Table2.DOCX]

Supplementary Table 2RNA Quantification and Quality Assurance by NanoDrop ND-1000

| SampleID | OD260/280 Ratio | OD260/230 Ratio | Conc.(ng/μl) | Volume(μl) | Quantity(ng) |
| --- | --- | --- | --- | --- | --- |
| P1 | 1.70 | 0.65 | 40.29 | 10 | 402.9 |
| P2 | 1.46 | 0.73 | 33.20 | 10 | 332.0 |
| P3 | 1.70 | 0.65 | 30.41 | 10 | 304.1 |
| P4 | 1.46 | 0.73 | 34.04 | 10 | 340.4 |
| P5 | 1.70 | 0.65 | 29.89 | 10 | 298.9 |
| P6 | 1.46 | 0.73 | 31.05 | 10 | 310.5 |
| P7 | 1.70 | 0.65 | 31.42 | 10 | 314.2 |
| P8 | 1.46 | 0.73 | 28.52 | 10 | 285.2 |
| P9 | 1.70 | 0.65 | 30.42 | 10 | 304.2 |
| P10 | 1.46 | 0.73 | 26.92 | 10 | 269.2 |
| P11 | 1.70 | 0.65 | 26.96 | 10 | 269.6 |
| P12 | 1.46 | 0.73 | 28.65 | 10 | 286.5 |
| P13 | 1.70 | 0.65 | 21.80 | 10 | 218.0 |
| P14 | 1.46 | 0.73 | 24.84 | 10 | 248.4 |
| P15 | 1.70 | 0.65 | 24.39 | 10 | 243.9 |
| P16 | 1.46 | 0.73 | 21.74 | 10 | 217.4 |
| P17 | 1.70 | 0.65 | 49.68 | 10 | 496.8 |
| P18 | 1.46 | 0.73 | 20.39 | 10 | 203.9 |
| P19 | 1.70 | 0.65 | 29.56 | 10 | 295.6 |
| P20 | 1.46 | 0.73 | 29.02 | 10 | 290.2 |
| P21 | 1.70 | 0.65 | 28.77 | 10 | 287.7 |
| P22 | 1.46 | 0.73 | 27.15 | 10 | 271.5 |
| H1 | 1.72 | 0.39 | 47.82 | 10 | 478.2 |
| H2 | 1.65 | 0.53 | 31.19 | 10 | 311.9 |
| H3 | 1.59 | 0.62 | 29.59 | 10 | 295.9 |
| H4 | 1.56 | 0.37 | 50.28 | 10 | 502.8 |
| H5 | 1.50 | 0.36 | 30.56 | 10 | 215.0 |
| H6 | 1.15 | 0.30 | 30.77 | 10 | 307.7 |
| H7 | 1.68 | 0.25 | 32.86 | 10 | 32.0 |
| H8 | 1.71 | 0.47 | 29.45 | 10 | 294.5 |
| H9 | 1.43 | 0.43 | 29.84 | 10 | 298.4 |
| H10 | 1.69 | 0.35 | 32.19 | 10 | 321.9 |
| H11 | 1.52 | 0.49 | 27.31 | 10 | 273.1 |
| H12 | 1.61 | 0.23 | 23.29 | 10 | 232.9 |
| H13 | 1.53 | 0.42 | 28.51 | 10 | 285.1 |
| H14 | 1.78 | 0.61 | 21.46 | 10 | 214.6 |
| H15 | 1.64 | 0.49 | 20.51 | 10 | 205.1 |
| H16 | 1.50 | 0.41 | 21.47 | 10 | 214.7 |
| H17 | 1.58 | 0.45 | 20.70 | 10 | 207.0 |
| H18 | 1.68 | 0.55 | 21.66 | 10 | 216.6 |
| H19 | 1.73 | 0.38 | 24.56 | 10 | 245.6 |
| H20 | 1.52 | 0.38 | 29.32 | 10 | 293.1 |
| H21 | 1.51 | 0.32 | 21.47 | 10 | 214.7 |
| H22 | 1.60 | 0.32 | 36.62 | 10 | 366.2 |
