# Supplementary material for: Physiological Adjustments and Circulating MicroRNA Reprogramming Are Involved in Early Acclimatization to High Altitude in Chinese Han Males
Source: Front Physiol. 2016 Dec 2;7:601. doi: 10.3389/fphys.2016.00601 (PMC5133430; doi:10.3389/fphys.2016.00601)
Supplement: Supplementary file 3 [file Table3.DOCX]

Supplementary Table 3 Paired T-Test or Wilcoxon Signed-rank Test Results of 33 Phenotypes between Lhasa and The Departure

| Phenotype | n | Pre-value | Post-value | Direction |  | T/Z Value | P Value | Reference | P |
| --- | --- | --- | --- | --- | --- | --- | --- | --- | --- |
| NOR^*^ | 22 | 947.3(210.66) | 828.4(161.80) | ↓ |  | 2.226 | 0.037 | <1700 | P<0.05 |
| ADR^#^ | 22 | 104.9(19.14) | 121.0(37.28) |  |  | -1.412 | 0.158 |  |  |
| DOP^#^ | 22 | 97.5(29.26) | 91.4(48.04) |  |  | -0.081 | 0.935 |  |  |
| F^#^ | 22 | 441.0(192.50) | 524.0(121.20) | ↑ |  | -2.841 | 0.005 | 138-690 | P<0.01 |
| ACTH^#^ | 22 | 22.4(19.38) | 26.1(18.53) |  |  | -1.120 | 0.263 |  |  |
| UA^*^ | 22 | 355.5(72.22) | 346.8(55.56) |  |  | 0.914 | 0.371 |  |  |
| CR^*^ | 22 | 83.1(11.65) | 91.9(12.98) | ↑ |  | -6.646 | 0.000 | 62-106 | P<0.001 |
| BUN^#^ | 22 | 4.8(1.35) | 4.3(1.05) |  |  | -0.829 | 0.407 |  |  |
| TP^*^ | 22 | 77.6(5.06) | 81.5(5.97) | ↑ |  | -2.419 | 0.025 | 65-85 | P<0.05 |
| ALB^#^ | 22 | 50.4(2.88) | 49.8(4.52) |  |  | -0.698 | 0.485 |  |  |
| GLB^*^ | 22 | 27.9(3.76) | 31.6(2.99) | ↑ |  | -3.393 | 0.003 | 20-40 | P<0.01 |
| A/G^*^ | 22 | 1.8(0.23) | 1.6(0.11) | ↓ |  | 4.587 | 0.000 | 1.2-2.4 | P<0.001 |
| TBL^#^ | 22 | 13.5(5.7) | 18.4(7.72) | ↑ |  | -3.442 | 0.001 | 0-17.1 | P<0.01 |
| DBL^*^ | 22 | 6.0(2.86) | 7.1(2.64) |  |  | -2.057 | 0.052 |  |  |
| IBIL^#^ | 22 | 8.2(2.90) | 12.3(4.17) | ↑ |  | -3.162 | 0.002 | 0-13.7 | P<0.01 |
| AST^#^ | 22 | 19.0(9.75) | 19.0(8.25) |  |  | 0.000 | 1.000 |  |  |
| ALT^#^ | 22 | 17.0(3.75) | 15.5(4.75) |  |  | -1.699 | 0.089 |  |  |
| R-GT^#^ | 22 | 18.5(8.75) | 20.5(7.00) |  |  | -0.863 | 0.388 |  |  |
| ALP^#^ | 22 | 65.0(17.75) | 65.5(21.50) | ↑ |  | -3.162 | 0.002 | 45-125 | P<0.01 |
| AST/ALT^*^ | 22 | 1.2(0.44) | 1.3(0.42) |  |  | -0.732 | 0.472 |  |  |
| CRP^#^ | 22 | 0.5(0.38) | 0.5(0.40) |  |  | -0.166 | 0.868 |  |  |
| CK^#^ | 22 | 89.5(32.00) | 115.5(43.50) | ↑ |  | -3.231 | 0.001 | 20-200 | P<0.01 |
| LDH^*^ | 22 | 139.0(18.38) | 154.5(19.87) | ↑ |  | -4.338 | 0.000 | 135-225 | P<0.001 |
| CKMB^#^ | 22 | 12.0(5.00) | 12.0(5.00) |  |  | -1.282 | 0.200 |  |  |
| TCH^#^ | 22 | 3.7(1.07) | 4.3(0.87) | ↑ |  | -2.305 | 0.021 | 0-5.2 | P<0.05 |
| TG^*^ | 22 | 0.9(0.36) | 0.8(0.32) | ↓ |  | 2.603 | 0.017 | 0-2.26 | P<0.05 |
| HDLC^*^ | 22 | 1.3(0.27) | 1.1(0.31) | ↓ |  | 4.275 | 0.000 | >1.04 | P<0.001 |
| LDLC^*^ | 22 | 2.3(0.66) | 2.6(0.64) | ↑ |  | -3.320 | 0.003 | <3.37 | P<0.01 |
| WBC^*^ | 20 | 5.7(1.84) | 6.6(1.70) | ↑ |  | -6.038 | 0.000 | 4-10 | P<0.001 |
| RBC^*^ | 20 | 4.8(1.19) | 5.4(0.43) | ↑ |  | -2.607 | 0.017 | 4-5.5 | P<0.05 |
| HG^*^ | 20 | 149.9(9.28) | 163.9(11.32) | ↑ |  | -7.936 | 0.000 | 120-160 | P<0.001 |
| HCT^*^ | 20 | 45.2(3.16) | 48.7(3.34) | ↑ |  | -5.713 | 0.000 | 0.39-0.51 | P<0.001 |
| PLT^*^ | 20 | 210.9(37.68) | 210.2(46.10) |  |  | 0.086 | 0.932 |  |  |

*Normal distribution data presented as Mean(SD), Tested by Paired T-Test; # Un-normal distribution data presented as Median(IQR), Tested by Wilcoxon Signed-rank Test
