# Supplementary material for: Physiological Adjustments and Circulating MicroRNA Reprogramming Are Involved in Early Acclimatization to High Altitude in Chinese Han Males
Source: Front Physiol. 2016 Dec 2;7:601. doi: 10.3389/fphys.2016.00601 (PMC5133430; doi:10.3389/fphys.2016.00601)
Supplement: Supplementary file 4 [file Table4.DOCX]

Supplementary Table 4 Person Coefficient Correlation of Phenotypes-Circulating microRNA Expression

| Phenotypes | 0.5 ~ 1 | 0.3 ~ 0.5 | 0.3 ~ -0.3 | -0.3 ~ -0.5 | -0.5 ~ -1 | Total |
| --- | --- | --- | --- | --- | --- | --- |
| SBP | 3 | 2 | 0 | 0 | 0 | 5 |
| DBP | 5 | 7 | 0 | 0 | 0 | 12 |
| HR | 0 | 1 | 0 | 0 | 0 | 1 |
| SaO2 | 0 | 0 | 0 | 1 | 0 | 1 |
| NOR | 1 | 0 | 0 | 1 | 1 | 3 |
| F | 0 | 0 | 0 | 11 | 11 | 22 |
| CR | 0 | 0 | 0 | 0 | 2 | 2 |
| TP | 1 | 0 | 0 | 1 | 0 | 2 |
| GLB | 1 | 0 | 0 | 0 | 0 | 1 |
| A/G | 0 | 1 | 0 | 1 | 0 | 2 |
| TBL | 0 | 1 | 0 | 1 | 0 | 2 |
| IBIL | 0 | 1 | 0 | 0 | 0 | 1 |
| ALP | 1 | 3 | 0 | 3 | 1 | 8 |
| CK | 0 | 1 | 0 | 1 | 0 | 2 |
| LDH | 0 | 1 | 0 | 5 | 0 | 6 |
| TCH | 0 | 1 | 0 | 12 | 12 | 25 |
| TG | 0 | 0 | 0 | 1 | 1 | 2 |
| HDLC | 0 | 0 | 0 | 1 | 2 | 3 |
| LDLC | 1 | 1 | 0 | 14 | 16 | 32 |
| WBC | 2 | 1 | 0 | 0 | 0 | 3 |
| RBC | 2 | 1 | 0 | 0 | 0 | 3 |
| HG | 0 | 0 | 0 | 4 | 1 | 5 |
| HCT | 0 | 1 | 0 | 2 | 2 | 5 |
