# Supplementary material for: Physiological Adjustments and Circulating MicroRNA Reprogramming Are Involved in Early Acclimatization to High Altitude in Chinese Han Males
Source: Front Physiol. 2016 Dec 2;7:601. doi: 10.3389/fphys.2016.00601 (PMC5133430; doi:10.3389/fphys.2016.00601)
Supplement: Supplementary file 5 [file Table5.DOCX]

Supplementary Table 5 Independent T-Test or Mann-Whitney U Test Results of 33 Phenotype changes between acclimatized and un-acclimatized individuals

| Phenotype | n | T/Z Value | P Value |
| --- | --- | --- | --- |
| NOR^*^ | 22 | -0.585 | 0.565 |
| ADR^*^ | 22 | 0.259 | 0.798 |
| DOP^*^ | 22 | -0.524 | 0.606 |
| F^*^ | 22 | 0.500 | 0.623 |
| ACTH^*^ | 22 | -0.871 | 0.394 |
| UA^*^ | 22 | -1.629 | 0.119 |
| CR^*^ | 22 | 0.321 | 0.752 |
| BUN^*^ | 22 | 0.846 | 0.407 |
| TP^*^ | 22 | 0.083 | 0.935 |
| ALB^*^ | 22 | 0.064 | 0.950 |
| GLB^*^ | 22 | 0.091 | 0.928 |
| A/G^*^ | 22 | 0.049 | 0.961 |
| TBL^*^ | 22 | -1.186 | 0.250 |
| DBL^*^ | 22 | -0.894 | 0.382 |
| IBIL^*^ | 22 | -1.241 | 0.229 |
| AST^*^ | 22 | -0.330 | 0.745 |
| ALT^*^ | 22 | -1.038 | 0.312 |
| R-GT^*^ | 22 | 0.482 | 0.641 |
| ALP^*^ | 22 | 0.240 | 0.813 |
| AST/ALT^*^ | 22 | 0.709 | 0.487 |
| CRP^*^ | 22 | -0.752 | 0.461 |
| CK^#^ | 22 | -1.403 | 0.161 |
| LDH^*^ | 22 | -0.041 | 0.968 |
| CKMB^*^ | 22 | 1.261 | 0.222 |
| TCH^*^ | 22 | -0.787 | 0.441 |
| TG^#^ | 22 | -0.267 | 0.789 |
| HDLC^*^ | 22 | -0.065 | 0.949 |
| LDLC^*^ | 22 | -0.524 | 0.606 |
| WBC^*^ | 20 | 0.790 | 0.450 |
| RBC^#^ | 20 | -0.835 | 0.403 |
| HG^*^ | 20 | 0.267 | 0.792 |
| HCT^*^ | 20 | -0.078 | 0.939 |
| PLT^#^ | 20 | -0.067 | 0.947 |

* Tested by Independent T-Test; # Tested by Mann-Whitney U Test
