# Supplementary material for: Physiological Adjustments and Circulating MicroRNA Reprogramming Are Involved in Early Acclimatization to High Altitude in Chinese Han Males
Source: Front Physiol. 2016 Dec 2;7:601. doi: 10.3389/fphys.2016.00601 (PMC5133430; doi:10.3389/fphys.2016.00601)
Supplement: Supplementary file 6 [file Image1.PDF]

Physiological adjustments and circulating microRNAs reprogramming are involved in early acclimatization to high altitude in Chinese Han males

Bao Liu[1#], He Huang[1#], Shouxian Wang[1], Gang Wu[1], Gang Xu[1], Bingda Sun[1], Erlong Zhang[1], Yuqi Gao[1]

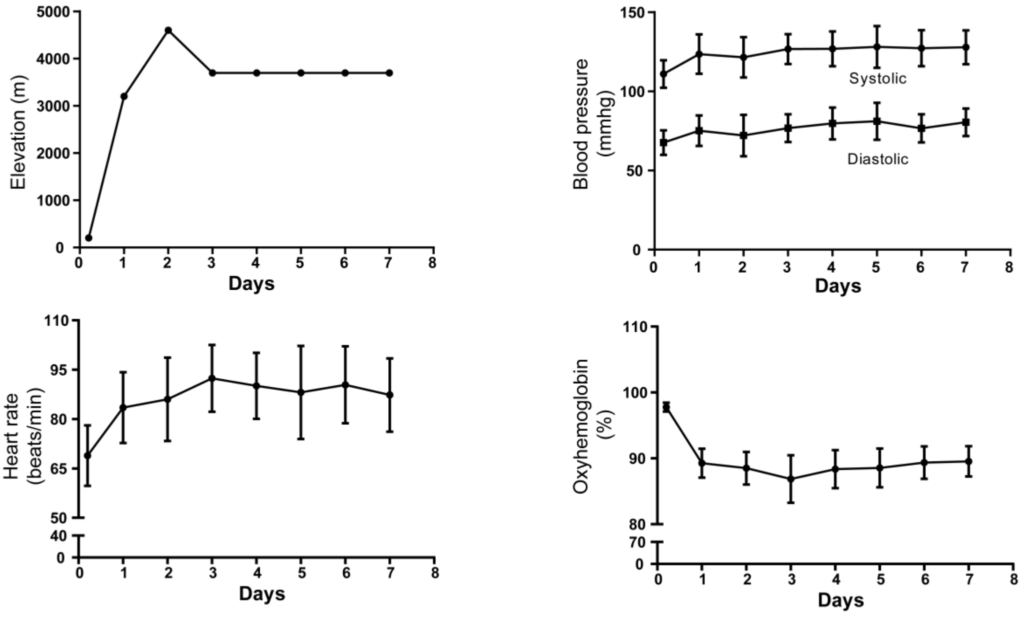

Supplementary Figure 1.

Ascent profile and altitude-related effects on cardio system as well as the delivery and consumption of oxygen during the exposure to high altitude. Arrows in top panel indicate location and timing of blood sampling. Data was shown as means ± SD; n = 22.

Hematological indices

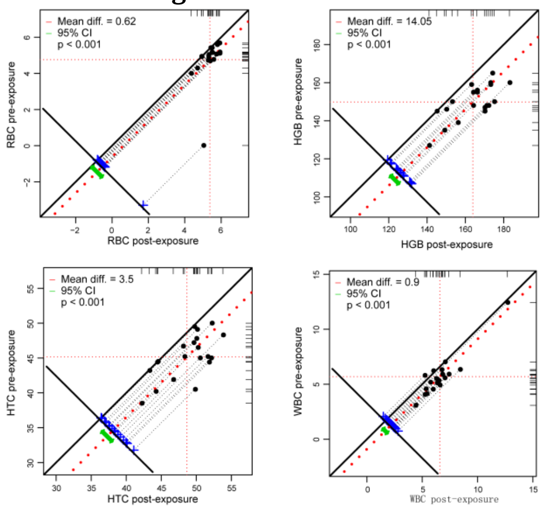

Myocardial enzyme spectrum

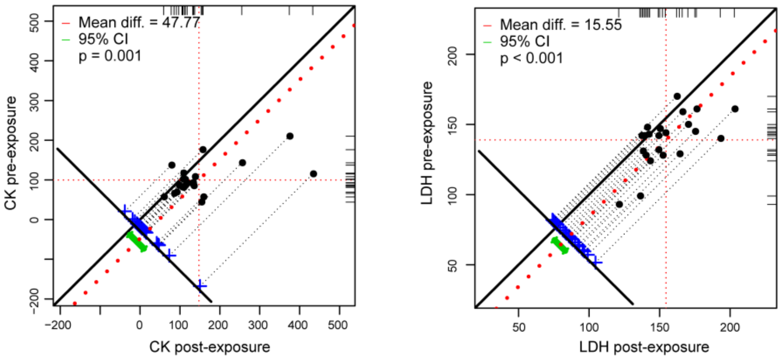

Enzymes related to liver and kidney functions

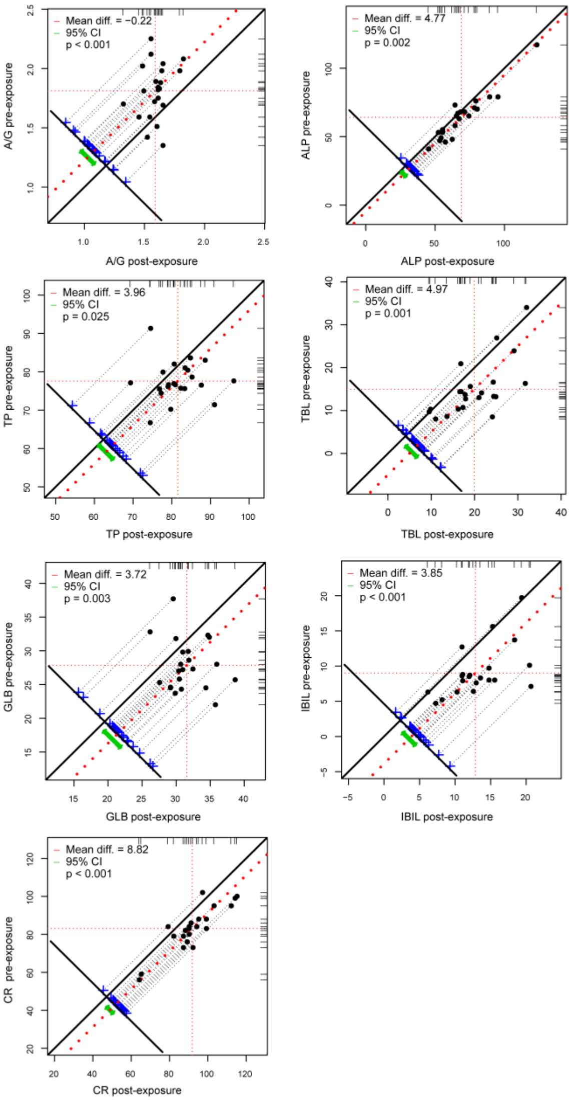

Supplementary Figure 2.

Ascent to high altitude is associated with adjustments of hematological indices, myocardial enzyme spectrum, and enzymes related to liver and kidney functions. n = 22.
